# Economic conditions, hypertension, and cardiovascular disease: analysis of the Icelandic economic collapse

**DOI:** 10.1186/s13561-017-0157-3

**Published:** 2017-05-23

**Authors:** Kristín Helga Birgisdóttir, Stefán Hrafn Jónsson, Tinna Laufey Ásgeirsdóttir

**Affiliations:** 1 Faculty of Economics, University of Iceland, Oddi v/Sturlugotu, 101 Reykjavik, Iceland; 2 Faculty of Social and Human Sciences, University of Iceland, Oddi v/Sturlugotu, 101 Reykjavik, Iceland

**Keywords:** Prolonged exposure, Crisis, Economic conditions, Economic downturn, Hypertension, Iceland

## Abstract

Previous research has found a positive short-term relationship between the 2008 collapse and hypertension in Icelandic males. With Iceland's economy experiencing a phase of economic recovery, an opportunity to pursue a longer-term analysis of the collapse has emerged. Using data from a nationally representative sample, fixed-effect estimations and mediation analyses were performed to explore the relationship between the Icelandic economic collapse in 2008 and the longer-term impact on hypertension and cardiovascular health. A sensitivity analysis was carried out with pooled logit models estimated as well as an alternative dependent variable. Our attrition analysis revealed that results for cardiovascular diseases were affected by attrition, but not results from estimations on the relationship between the economic crisis and hypertension. When compared to the boom year 2007, our results point to an increased probability of Icelandic women having hypertension in the year 2012, when the Icelandic economy had recovered substantially from the economic collapse in 2008. This represents a deviation from pre-crisis trends, thus suggesting a true economic-recovery impact on hypertension.

## Background

The link between business cycles and health has been studied to a considerable extent. The Great Recession has sparked interest and opportunities to pursue this line of research further. The Icelandic economic collapse is already established as a favorable treatment [1–13] due to the clear before and after contrast that results from a collapse that can be pinpointed almost to a specific date; October 6th 2008 when Iceland’s Prime Minister announced the risk of national bankruptcy [14]. Subsequently, the Icelandic economy contracted by 6.6% in 2009 and 4.1% in 2010 and was among the hardest hit in the world [15]. Thereafter, the Icelandic economy experienced substantial recovery, so much so, that it received international attention as one of Europe’s top performers [16–21].

**Fig. 1:**
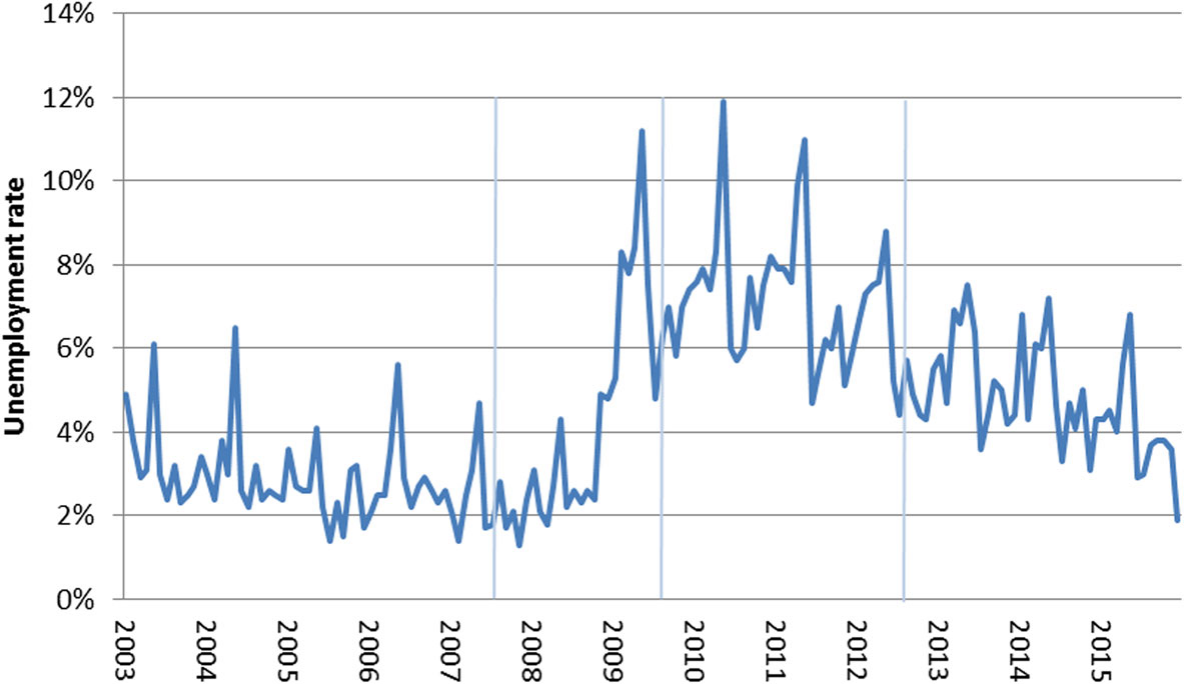
Monthly unemployment rate in Iceland January 2003-December 2015. Source: Statistics Iceland. Accessed October 17th 2016 from: http://px.hagstofa.is/pxen/pxweb/en/Samfelag/Samfelag__vinnumarkadur__vinnumarkadur/VIN00001.px/table/tableViewLayout1/?rxid = 07c0d8b2-a5b9-4bc0-a75c-a40e871fd831. Notes: Vertical lines refer to the timing of 1st, 2nd, and 3rd waves of data collection for the survey data used here

**Fig. 2:**
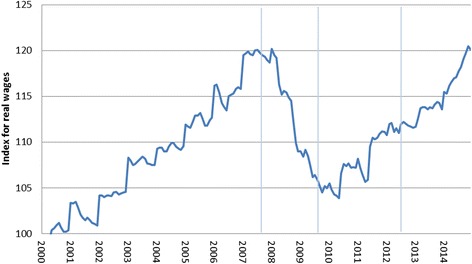
Monthly index for real wages in Iceland January 2003-December 2015. Source: Statistics Iceland. Accessed October 17th 2016 from: http://px.hagstofa.is/pxen/pxweb/en/Efnahagur/Efnahagur__thjodhagsreikningar__efnahagslegar_skammtimatolur/THJ00117.px/table/tableViewLayout1/?rxid = 07c0d8b2-a5b9-4bc0-a75c-a40e871fd831. Notes: Base level of 100 is set in the year 2000. The index refers to the change in the wage index deflated by the CPI converted to mid-month figures. Vertical lines refer to the timing of 1st, 2nd, and 3rd waves of data collection for the survey data used here

Up until the early 2000s, Iceland’s economy was export-driven, with fishing and aluminum smelting serving as the main industries, but after the deregulation of Icelandic banks the country’s financial sector expanded in a major way, with the three biggest commercial banks in Iceland growing to almost 10 times the size of the Icelandic economy. This led to a bubble that was primed to pop when international short-term funding dried up [22]. For a country the size of Iceland, with a population of 330,000, the impact of the economic collapse was widely felt; people’s savings vanished with the crash of the Icelandic stock market (of which the three biggest commercial banks comprised more than half of listed stocks) [23–25], monthly unemployment tripled and remained high compared to the pre-crisis long-term unemployment rate of around 2.5–3% [26] (Fig. 1), and real wages plummeted [27] (Fig. 2).

Not all medical conditions are theoretically likely to be affected by external factors, for example various genetic diseases. Cardiovascular events have however been shown to be responsive to such factors, for example stressful circumstances such as war [28, 29] and earthquakes [30–32], as well as important sporting events which might trigger emotional stress [33–37]. For this reason, cardiovascular outcomes have been of interest in the health and business cycle literature (see for example studies by Gerdtham and Ruhm [38], Ruhm [39], Ruhm [40], Ruhm [41], Neumayer [42], Tapia Granados and Ionides [43]). It seems a priori plausible that as large a business cycle event as the economic collapse in Iceland would affect hypertension and cardiovascular diseases in Icelanders. Although effects of business cycles on health could be the greatest at the extremes of the cycle, it is possible that some health effects take time to present themselves, as explored for total mortality in Ruhm [44] and Stuckler et al. [45], as well as other diseases, such as cardiovascular diseases in Gerdtham and Ruhm [38] and Ruhm [41]. The immediate effects of the Icelandic economic collapse on health have been studied to some extent, but a chance to examine longer-term effects for comparison is gradually emerging as time passes. We focus first and foremost on longer-term effects on hypertension, one of the most important risk factors for cardiovascular diseases [46–49] and the leading preventable risk factor for premature death worldwide [50, 51], but also examine cardiovascular disease for completeness, thus following up on a previously published analysis of short-term effects on those outcomes [7].

Both the short and longer-term impacts of economic conditions on cardiovascular disease are unclear, due to the multitude of determinants of cardiovascular health. Some are known to get more favorable during times of economic hardship, such as smoking and alcohol misuse [1–3, 6, 44, 52, 53] and others are known to become less favorable, such as psychological morbidity [5]. Similarly the determinants of cardiovascular health include both long-term determinants that make the individual more vulnerable, as well as short-time stressors [54–56], making it important to examine effects with a different time lag. Aside from the factors mentioned, unemployment and income are examined here as possible mediators in the relationship, as they have been found to be positively associated with cardiovascular mortality and morbidity [57–62], possibly through changed behavior and consumption patterns. As the literature has moved towards more detailed exploration of possible mechanisms underlying the relationships explored, we follow that direction in our mediation analysis. Overall, the empirical framework for our study stems from the pioneering work of Grossman [63, 64], as well as on the Grossman-derived demand for health behaviors as described in work by Xu and Kaestner [65].

The relationship between economic downturns and cardiovascular health is complicated and results have been mixed across settings (the interested reader is referred to the extensive supplementary online literary review by Asgeirsdottir et al. [7]). This study adds to the growing literature in various ways. Firstly, it does so by examining the longer-term effects in a follow-up study to Asgeirsdottir et al. [7], which looked into the short-term effects. Secondly, nationally representative, individual-level data are used, where the same Icelanders have partaken in a survey before and after the economic downturn, as opposed to aggregate data which have been dominant in the field. This allows us to study possible individual-level mediators in the relationship between the economic crisis and cardiovascular health, i.e. to assess the extent to which heterogeneously felt effects of the crisis explain the effects of the economic-recovery indicator. Similar to the way that Asgeirsdottir et al. [3] expanded on the short-term results of Asgeirsdottir et al. [2] on health behaviors, this paper expands on the short-term results of Asgeirsdottir et al. [7] on hypertension and cardiovascular diseases, largely following the methodology of those studies. Thirdly, an unusually comprehensive dataset on hypertension and cardiovascular morbidity is utilized, whereas the previous literature has, due to data restrictions, mostly studied mortality.

## Methods

The data used here is the lifestyle survey “Heilsa og líðan Íslendinga” (Health and well-being of the Icelandic population) carried out by the Icelandic Public Health Institute in 2007 and 2009 and then Icelandic Directorate of health in 2012, providing data from periods of economic boom, bust, and recovery. The survey contains questions regarding health and lifestyle, as well as demographics, labor participation, and income.

A stratified random sample of 9807 individuals 18–79 years old was drawn. In 2007 9711 individuals received questionnaires with a response rate of 60.9%, or 5909 returned questionnaires. The 2009-sample included 5294 of the original individuals who had agreed to be contacted again. For the 2009 survey the response rate was 69.3%, or 4092 individuals. In 2012 the sample of original participants who had agreed to be contacted for follow-up studies consisted of 3.659 individuals. The response rate was 88.5%, or 3238 individuals, corresponding to 33.0% of the original sample. Additionally, in 2012 a sample of 3506 new subjects was added. The sampling method for the new entrants was comparable to the ones of the original sample in 2007, thus providing cross-sectional data across 2007 and 2012, in addition to the panel of same individuals answering in those years. In our main analysis using fixed-effect models and in the sensitivity analysis using panel data we use a balanced panel of only those who answered questionnaires from all three years. Answers from the new participants in 2012 were however only used in an alternative analysis (results found in the supplementary online material) where the cross-sectional aspects of the data were taken advantage of.

We perform two analyses, using the panel data. Methodologically, each one has its pros and cons, but together they provide a more comprehensive picture than each individual method. For our main analysis we estimate individual fixed-effects models, as is frequently done when panel data are available. These models implicitly control for all unobserved time-invariant individual heterogeniety. Additionally, they account for cross-period correlation in standard errors. An argument against using fixed-effects models in our analyses is a possible bias in the measurement of the coefficient we are most interested in measuring, the recovery indicator, as reported and explained in Asgeirsdottir et al. [7] with a detailed mathematical rationalization of the choice for a pooled model in their supplementary online material. Their explanation applies here as well. Therefore, in our sensitivity analysis we perform an additional analysis, with pooled logit models. In addition, we use a different variable to gauge health of participants, i.e. the use of prescription medication. One would expect the correlation between a diagnosis of a disease and the use of prescription medication for that disease to be high, but in our data, that is not the case; the highest correlation coefficient found in the data is 0.658 for hypertension. Hence, we feel that a sensitivity analysis using prescription medication as a proxy for health is in order. Furthermore, we perform an attrition analysis to address the concern of possible attrition bias (results available in the supplementary online material).

Due to deliberate oversampling of older age groups and those living outside the capital area, sample weights are included in all estimations. When sample weights are used, the sample is representative of the Icelandic population in 2007 [66].

In Tables 1 and 2 unadjusted summary statistics are reported for males and females in the full panel data sample. To inspect the statistical significance of the differences in each variable between waves, t-tests were carried out and corresponding *p*-values reported in the same tables. The summary statistics only represent the raw data for participants in the final sample and do not expose any crisis effect since important factors have not been controlled for.

**Table 1:** Full panel data sample summary statistics: males answering both waves

	2007			2012			*t*-test
Variable	Mean	SD	N	Mean	SD	N	*p*-value
Age	55.075	14.893	1501	60.075	14.893	1501	0.0000
1 if hypertension	0.218	0.413	1460	0.259	0.438	1438	0.0086
1 if coronary thrombosis	0.019	0.137	1458	0.030	0.171	1431	0.0598
1 if coronary disease	0.029	0.168	1451	0.051	0.221	1422	0.0022
1 if stroke	0.004	0.064	1466	0.004	0.065	1433	0.9685
1 if cardiovascular disease	0.041	0.197	1502	0.061	0.239	1502	0.0125
No. of children	2.594	1.563	1482	2.692	1.533	1490	0.0840
1 if rural	0.382	0.486	1494	0.387	0.487	1487	0.7436
1 if married	0.703	0.457	1479	0.707	0.455	1472	0.8107
1 if cohabiting	0.124	0.330	1479	0.117	0.321	1472	0.5285
1 if single or in a relationship	0.116	0.320	1479	0.094	0.292	1472	0.0524
1 if divorced	0.035	0.184	1479	0.043	0.202	1472	0.2837
1 if widowed	0.022	0.146	1479	0.039	0.195	1472	0.0050
1 if educ1^a^	0.295	0.456	1498	0.295	0.456	1498	
1 if educ2^a^	0.292	0.455	1498	0.292	0.455	1498	
1 if educ3^a^	0.182	0.386	1498	0.182	0.386	1498	
1 if educ4^a^	0.138	0.345	1498	0.138	0.345	1498	
1 if educ5^a^	0.091	0.288	1498	0.091	0.288	1498	
1 if hypertension medication	0.219	0.414	1440	0.310	0.463	1447	0.0000
1 if cholesterol medication	0.148	0.355	1454	0.224	0.417	1466	0.0000
1 if circulatory disease medication	0.054	0.226	1465	0.068	0.252	1463	0.1033
Body Max Index (BMI)	27.410	4.243	1472	27.506	3.808	1478	0.5194
1 if underweight	0.003	0.052	1472	0.002	0.045	1478	0.7012
1 if optimal weight	0.272	0.445	1472	0.262	0.440	1478	0.5435
1 if overweight	0.519	0.500	1472	0.520	0.500	1478	0.9447
1 if obese	0.207	0.405	1472	0.216	0.412	1478	0.5358
1 if non smoker	0.829	0.377	1468	0.876	0.330	1470	0.0004
1 if daily smoker	0.137	0.344	1468	0.091	0.288	1470	0.0001
1 if weekly smoker	0.016	0.124	1468	0.017	0.129	1470	0.7747
1 if seldom smoker	0.018	0.134	1468	0.016	0.127	1470	0.6683
1 if non drinker	0.114	0.318	1469	0.144	0.351	1475	0.0149
1 if daily drinker	0.028	0.165	1469	0.029	0.168	1475	0.8396
1 if frequent drinker	0.338	0.473	1469	0.296	0.457	1475	0.0142
1 if seldom drinker	0.378	0.485	1469	0.372	0.483	1475	0.6965
1 if rare drinker	0.142	0.349	1469	0.159	0.366	1475	0.1787
Perceived Stress Scale (PSS)	7.154	1.757	1446	7.468	1.695	1428	0.0000
1 if unemployed	0.027	0.161	1457	0.037	0.189	1449	0.1079
1 if much better financial status^b^	0.017	0.128	1446	0.017	0.128	1440	0.9884
1 if considerably better financial status^b^	0.126	0.332	1446	0.120	0.325	1440	0.6397
1 if somewhat better financial status^b^	0.214	0.410	1446	0.210	0.408	1440	0.8296
1 if similar financial status^b^	0.508	0.500	1446	0.501	0.500	1440	0.6830
1 if somewhat worse financial status^b^	0.082	0.275	1446	0.095	0.294	1440	0.2251
1 if considerably worse financial status^b^	0.036	0.186	1446	0.042	0.200	1440	0.4277
1 if much worse financial status^b^	0.017	0.130	1446	0.014	0.117	1440	0.4612
Real income^c^	5.869	3.136	1445	4.558	2.340	1449	0.0000
Work hours	5.815	4.868	1386	4.976	4.844	1327	0.0000

Summary statistics only represent the data and do not display any crisis effect. Means are unweighted. *P*-values are from *t*-test for differences in means between 2007 and 2012

^a^Education level is represented by dummy variables: educ1 represents primary or lower level secondary education; educ2 stands for vocational master or journeyman certificate; educ3 stands for high school or equivalent; educ4 stands for technical graduate or undergraduate degree; educ5 stands for master’s degree or a Ph.D

^b^Perceived financial status represents respondents’ own perception of their families’ financial status relative to other families

^c^Real income is reported at 2012 price level in millions of Icelandic kronas (ISK)

**Table 2:** Full panel data sample summary statistics: females answering both waves

	2007			2012			*t*-test
Variable	Mean	SD	N	Mean	SD	N	*p*-value
Age	52.057	16.190	1736	57.057	16.190	1736	
1 if hypertension	0.234	0.423	1690	0.293	0.455	1667	0.0001
1 if coronary thrombosis	0.005	0.073	1688	0.009	0.092	1646	0.2685
1 if coronary disease	0.016	0.126	1674	0.026	0.160	1631	0.0411
1 if stroke	0.002	0.049	1683	0.004	0.060	1645	0.5033
1 if cardiovascular disease	0.019	0.137	1736	0.030	0.171	1736	0.0369
No. of children	2.572	1.550	1727	2.701	1.468	1717	0.0121
1 if rural	0.355	0.479	1704	0.347	0.476	1689	0.6214
1 if married	0.591	0.492	1711	0.584	0.493	1705	0.6647
1 if cohabiting	0.151	0.358	1711	0.129	0.335	1705	0.0669
1 if single or in a relationship	0.120	0.326	1711	0.104	0.305	1705	0.1246
1 if divorced	0.061	0.239	1711	0.070	0.256	1705	0.2573
1 if widowed	0.077	0.267	1711	0.113	0.317	1705	0.0003
1 if educ1^a^	0.454	0.498	1730	0.454	0.498	1730	
1 if educ2^a^	0.034	0.182	1730	0.034	0.182	1730	
1 if educ3^a^	0.210	0.408	1730	0.210	0.408	1730	
1 if educ4^a^	0.218	0.413	1730	0.218	0.413	1730	
1 if educ5^a^	0.083	0.275	1730	0.083	0.275	1730	
1 if hypertension medication	0.219	0.414	1672	0.293	0.455	1676	0.0000
1 if cholesterol medication	0.078	0.268	1689	0.134	0.341	1692	0.0000
1 if circulatory disease medication	0.017	0.130	1688	0.030	0.170	1679	0.0157
Body Max Index (BMI)	27.278	5.378	1683	27.448	4.988	1691	0.3419
1 if underweight	0.005	0.073	1683	0.006	0.077	1691	0.8262
1 if optimal weight	0.380	0.485	1683	0.352	0.478	1691	0.1008
1 if overweight	0.358	0.479	1683	0.374	0.484	1691	0.3333
1 if obese	0.257	0.437	1683	0.268	0.443	1691	0.4839
1 if non smoker	0.803	0.398	1676	0.853	0.355	1690	0.0001
1 if daily smoker	0.157	0.364	1676	0.116	0.320	1690	0.0005
1 if weekly smoker	0.015	0.121	1676	0.014	0.116	1690	0.7492
1 if seldom smoker	0.026	0.158	1676	0.018	0.132	1690	0.1155
1 if non drinker	0.155	0.362	1697	0.180	0.384	1692	0.0542
1 if daily drinker	0.015	0.123	1697	0.009	0.097	1692	0.1229
1 if frequent drinker	0.199	0.399	1697	0.180	0.384	1692	0.1598
1 if seldom drinker	0.402	0.491	1697	0.391	0.488	1692	0.4823
1 if rare drinker	0.229	0.420	1697	0.241	0.428	1692	0.4136
Perceived Stress Scale (PSS)	7.374	2.009	1668	7.852	1.705	1640	0.0000
1 if unemployed	0.293	0.169	1671	0.033	0.179	1684	0.5134
1 if much better financial status^b^	0.010	0.101	1641	0.011	0.102	1618	0.9670
1 if considerably better financial status^b^	0.090	0.286	1641	0.092	0.289	1618	0.8032
1 if somewhat better financial status^b^	0.190	0.392	1641	0.193	0.395	1618	0.7756
1 if similar financial status^b^	0.521	0.500	1641	0.498	0.500	1618	0.1916
1 if somewhat worse financial status^b^	0.124	0.329	1641	0.143	0.350	1618	0.1093
1 if considerably worse financial status^b^	0.052	0.223	1641	0.047	0.212	1618	0.4755
1 if much worse financial status^b^	0.012	0.110	1641	0.015	0.121	1618	0.5131
Real income^c^	3.799	2.446	1647	3.389	1.898	1629	0.0000
Work hours	4.514	4.119	1592	4.085	4.127	1569	0.0035

Summary statistics only represent the data and do not display any crisis effect. Means are unweighted. *P*-values are from *t*-test for differences in means between 2007 and 2012

^a^Education level is represented by dummy variables: educ1 represents primary or lower level secondary education; educ2 stands for vocational master or journeyman certificate; educ3 stands for high school or equivalent; educ4 stands for technical graduate or undergraduate degree; educ5 stands for master’s degree or a Ph.D

^b^Perceived financial status represents respondents’ own perception of their families’ financial status relative to other families

^c^Real income is reported at 2012 price level in millions of Icelandic kronas (ISK)

### Dependent variables

The dependent variables used are: hypertension; coronary thrombosis; coronary disease; stroke; and cardiovascular disease, and a binary variable indicating whether participants had any cardiovascular disease (CVD), i.e. coronary thrombosis, coronary disease, or stroke. Hypertension is the main outcome variable, but following Asgeirsdottir et al. [7] variables regarding cardiovascular health were included for completeness although the low number of observations for those outcomes leads to unreliable results. The response options in 2007 and 2009 were: “yes, have got it now”; “have had it before but not now”; “no, have never had it”. In the 2012 survey the response categories where changed so they became: “yes, have got it now”; “do not have it now, but had it within the last 12 months”; “do not have it now, but had it more than 12 months ago”; “no, have never had it”. If respondents answered “yes, have got it now”, they were also asked if a doctor had diagnosed them with the medical condition in question. A binary variable for the outcomes was constructed, taking into account the altered answering arrangement between waves, taking the value 1 if respondents marked both “yes, have got it now” for the relevant cardiovascular condition and if the medical condition in question was diagnosed by a doctor, but 0 otherwise. Due to few observations of coronary thrombosis, coronary disease, and stroke, a binary variable was created indicating if an individual reported having any cardiovascular disease (coronary thrombosis, coronary disease, or stroke). As can be seen in the summary statistics in Tables 1 and 2, the difference between 2007 and 2012 in the prevalence of both hypertension and cardiovascular diseases, is statistically significant for both genders.

In the sensitivity analysis we use binary variables for participants’ prescription medication use as dependent variables. Responses to questions on medication use for both hypertension as well as cardiovascular and cholesterol diseases were used. The variables take the value 1 if respondents answered positively to having taking such medication in the last 2 weeks, but 0 otherwise.

**Fig. 3:**
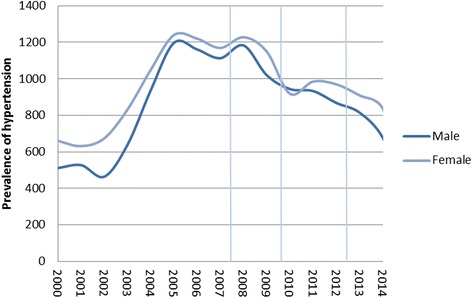
Prevalence of hypertension among patients with cardiovascular diseases in January 2000-December 2014. Source: Landspitali University Hospital. Notes: Vertical lines refer to the timing of 1st, 2nd, and 3rd waves of data collection for the survey data used here. The prevalence of hypertension is reported in absolute terms

**Fig. 4:**
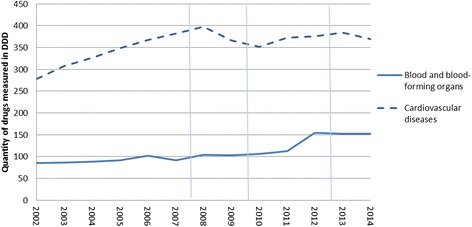
Usage for medication for cardiovascular diseases and blood and blood-forming organs in 2000-2014. Source: Statistics Iceland. February 6th 2017 from: http://px.hagstofa.is/pxen/pxweb/en/Samfelag/Samfelag__heilbrigdismal__heilbrigdisthjonusta/HEI08101.px/table/tableViewLayout1/?rxid = b55fddaf-5d24-4e01-885f-299513510b32. Notes: Vertical lines refer to the timing of 1st, 2nd, and 3rd waves of data collection for the survey data used here. The quantity of drugs is shown in defined daily dose (DDD) per 1000 inhabitants. DDD is according to WHO standard of each year

**Fig. 5:**
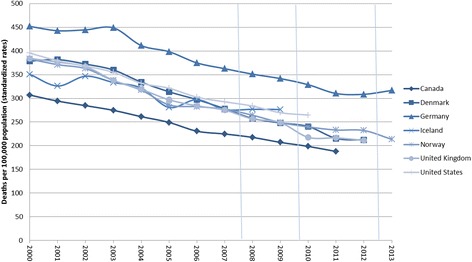
Aggregate standardized mortality rates due to circulatory diseases in 2000-2013. Source: OECD. August 11th 2015 from: http://stats.oecd.org/index.aspx?DataSetCode = HEALTH_STAT. Notes: Vertical lines refer to the timing of 1st, 2nd, and 3rd waves of data collection for the survey data used here

As we follow subjects over time in this analysis, pre-existing trends in the health outcomes present a potential methodological challenge in our study. Data on trends in hypertension and cardiovascular morbidity in Iceland is not available, but in an attempt to take this informally into account, figures from Iceland relating to the prevalence of these medical conditions were inspected, in addition to our sensitivity analyses using participants’ use of prescription medication as dependent variables. Specifically, aggregate data from Landspitali University Hospital from 2000 to 2014 on the prevalence of hypertension in all patients suffering from cardiovascular diseases were inspected (Fig. 3), as well as consumption of drugs for the blood and blood-forming organs, and cardiovascular system in Iceland [67] (Fig. 4). Furthermore, aggregate mortality rates due to circulatory diseases in Iceland and other countries [68] were examined (Fig. 5). Research on current and predicted hypertension prevalence is available for other countries, which find generally unchanged prevalence in most countries, although awareness and treatment of the disease is improving, thus leading to better hypertension outcomes [69–74]. A similar pattern is found between males and females when examining the prevalence of patients suffering from cardiovascular diseases, but not a specific time trend over the period as a whole (see Fig. 3). From 2000 to 2005 a near doubling of the prevalence (in absolute terms) is found followed by a substantial decline after the economic collapse in 2008. A clear upward trend in usage of the drugs is evident in the years and decades prior to the crisis. In the case of cardiovascular drugs, a peak was reached in 2008, followed by a rather steep decline until 2011, and during the economic recovery usage levelled off. Usage of drugs for blood and blood-forming organs was relatively even in the boom years (2004-2007) and during the crisis (2008–2010), with an increase during the economic recovery (2011–2012) (see Fig. 4). Mortality rates due to circulatory diseases are distinctly downward trending both before and throughout the study period (see Fig. 5); a similar trend can be seen in other Western countries which experienced the Great Recession to varying degrees (UK, USA, Germany, Norway, and Denmark). Although these numbers do not represent the impact of the economic crisis and recovery on hypertension and cardiovascular diseases, they do provide a context to interpret our results. This context is important as we are examining a single economic fluctuation. Although that fluctuation presents an important research opportunity, due to the exceptionally large changes in conditions over a short time period that are likely to overshadow other societal events occurring at the same time, we cannot rule out that normal fluctuations affect the results with this research design.

### Control variables

Depending on estimation model, either only age squared or both age and age squared are used as controls in continuous form. Five dummy variables are used for marital status; married; cohabitating; divorced; widowed; and single or in a non-cohabitating or non-marital relationship, which is used as the benchmark variable for marital status. A variable for the number of children was used in continuous form. A binary variable indicates whether an individual lived in an urban (an area of more than 5000 inhabitants) or rural area. As can be seen in Tables 1 and 2 demographics are relatively stable across waves.

Answering options were added on the questionnaire in 2009 regarding the education of respondents, and therefore it is not clear that changed answers between 2007 and 2009 reflect added education during that time, or that respondents found a more suitable answering option that fitted their educational status. Due to the greater detail in answering options a new time-invariant variable for education was constructed using the educational level in 2012 as a base, but imputations from 2009 to 2007 were used when answers were missing. Owing to the increased clarity of the educational question this is deemed the best option, and justified as variability in education is small over such a short time span. Education is thus rather being used as a control for a wider reaching social status. However, this variable is only used in the logit regression since a time-invariant variable cannot be included in the fixed-effects models. As the fixed-effects capture inherently what our education variable measures, it can be emitted from the fixed-effects models without harming the analysis. Five dummy variables were constructed for education; *educ1* represents those who finished primary or lower level secondary education and is use as benchmark in the analyses; *educ2* those who finished a vocational master or journeyman certificate; *educ3* those who finished high school or equivalent; *educ4* those who finished a technical graduate or undergraduate degree; and *educ5* those who had finished a master’s degree or a Ph.D.

### Exposure

Exposure to certain economic conditions is measured with time indicators. Due to the follow-up nature of this study, as an expansion of previous work on the short term effects of the collapse, the key independent variable *t2012* is a dummy for the time of the economic recovery during the third wave of data collection. Additionally, the time variable *t2009* captures the short-term exposure of the participants in our sample, but as noted earlier, the short-term impact on hypertension and cardiovascular disease was previously reported on in the literature [7]. Both variables, *t2009* and *t2012,* take the value 1 for the respective years, but zero otherwise. By including both time variables in all estimations, the year 2007 is used as a reference against the short-term and longer-term exposure of participants to the economic crisis.

### Mediators

The purpose of the mediation analysis is to attempt to disentangle the individual-level impact of the crisis on the possible mediating factors rather than to obtain unbiased estimates of the impact of each pathway. As this is an extension of previous work by Asgeirsdottir et al. [7], we conduct our mediation analysis in a similar way. The body mass index (BMI) is used to proxy overall body composition. BMI is calculated by dividing an individual’s weight in kilograms by the square of their height in meters. Four dummy variables corresponding to the four BMI categories were constructed (<18.5 is underweight; 18.5–24.9 is optimal and used as the benchmark; 25–29.9 is overweight; ≥30 indicates obesity). Four dummy variables representing smoking behaviors are: *daily smoker*; *weekly smoker*; *seldom smoker* for those who report smoking less than once a week; *non-smoker* is the benchmark for smoking behavior. While the unadjusted t-tests in Tables 1 and 2 show mostly a non-significant statistical difference between years in health behaviors, i.e. smoking and alcohol consumption, a reduction in daily smokers and increase in non-smokers for both genders is notable.

Five dummy variables represent alcohol consumption in the last 12 months. The variables are: *daily drinker*; *frequent drinker* for those who answered to having at least one drink 1–4 times a week; *seldom drinker* for those who answered having a drink 1–3 times a month or at least one drink 7–11 times in the last 12 months; *rare drinker* stands for those who had a drink 1–6 times in the last 12 months; *non-drinker* for those not having had an alcoholic drink in the last 12 months is the benchmark variable for alcohol consumption.

A short form of the Perceived Stress Scale (PSS) was used, consisting of four questions that measure to which degree situations in one’s life are conceived as stressful [75]. Five answering options for each question, translate to an overall score range from 0 to 16, with 16 representing the highest level of stress. Unadjusted stress levels increased significantly between the years for both genders (Tables 1 and 2).

The variable *work hours* refers to time spent on paid work. A question on hours spent each week on paid work had thirteen categories, ranging from 0 to over 60 hours per week. The midpoint of each category was used to ease readability of the estimated coefficients of work hours; the variable was scaled to working hours per day (assuming 5 working days per week) in continuous form. A dummy variable for unemployment was also used.

The variable *annual income* refers to the respondents’ complete income before taxes. Ten answering options were available, from less than 900 thousand Icelandic kronas (ISK) annually to more than 8.4 million ISK annually. The midpoint of each category was used as continuous, with a top of 9.0 million ISK used for the highest category. Inflation between the years 2007 and 2009 was 27.05%, between 2007 and 2012 it was 42.73%. Amounts were set to the 2012 price level. Real income decreased by a statistically significant amount between 2007 and 2012 for both males and females.

Seven dummy variables were constructed from the equal amount of response categories for perceived relative financial status in society based on answers to the question “In a financial sense, how well or badly off do you consider your family to be relative to other families in Iceland?” A perceived similar financial status relative to other Icelandic families is used as the benchmark variable in the mediation analysis.

### Estimations

In our main analysis, fixed-effects models are used to estimate the relationship between the timing of responses and the dependent variables, with the recovery indicator *t2012* capturing the impact of the economic recovery compared to the pre-crisis. As fixed-effects logit models did not suit the data due to a big proportion of the sample having no within-individual variation leading to many observations being dropped, we estimate linear probability fixed-effects models instead, using the estimation equation:

1 $$ {H}_{i t}=\alpha + t{2012}_{i t}{\beta}_1+ t{2009}_{i t}{\beta}_2+{X}_{i t}{\beta}_3+{M}_{i t}{\beta}_4+{v}_i+{e}_{i t} $$

Where *α* is a constant term, *H* is a health outcome for individual *i* at time *t*, *t2012* and *t2009* are indicators for long-term and short-term exposure to the economic crisis, making *β*_*1*_ our main coefficient of interest, *X* contains demographic variables including age, marital status, number of children, and residency, *M* are possible mediating factors that are only included in the mediation analysis, *v* is a term for individual fixed effects, and *e* is the disturbance term.

In the sensitivity analysis, pooled logit models are estimated. Similar to our main analysis the key variable is the recovery indicator, *t2012.* Results are reported as marginal effects calculated after logit regressions and all analyses are performed separately for males and females. To account for individual heteroscedasticity, standard errors are clustered on individuals in the logit regressions.

In the mediation analysis, one possible mediating factor was added at a time to the base models in order to assess the extent to which changes in each factor can explain changes in the recovery indicator, being observant of both mediating and possibly suppressing roles of those variables in the causal path between the independent and dependent variable [76, 77]. In an alternate mediation model, an interaction term of the possible mediating factor and the recovery indicator was also included in the mediation analysis to account for the possibility of moderated mediation, i.e. that the strength of the mediated relationship is contingent on the value of the recovery indicator [78–80]. For the sake of brevity, the results from the alternate mediation model are only included in the supplementary online material. Mediation tests using uncontrolled models were performed to test the significance of mediators. A Sobel-Goodman test [81] was performed, both with and without alterations to fit logit models, as described by Mackinnon and Dwyer [82], yielding almost identical results.

Stata 13.1 was used for all statistical computations. The study was approved by the Directorate of Health (1311268/5.6.1/gkg), the Ethics Board of Iceland (07-081, 09-094 and 12-107) and the Data Protection Authority of Iceland (S4455).

## Results

**Fig. 6:**
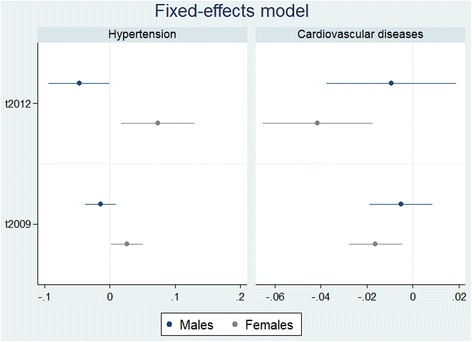
Regression results from fixed-effect models. Notes: Markers refer to point estimates from regressions. Horizontal lines refer to 90% confidence intervals

**Fig. 7:**
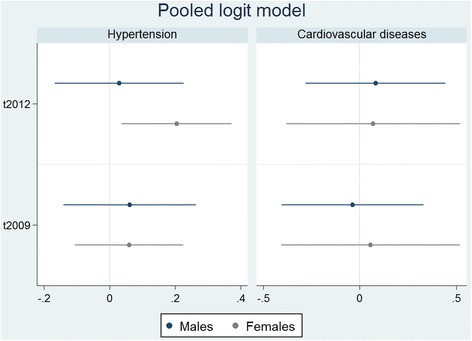
Regression results from pooled logit models. Notes: Markers refer to point estimates from regressions. Horizontal lines refer to 90% confidence intervals

Very few observations of coronary thrombosis, coronary disease, and stroke led to imprecise estimates which did not show statistically significant effects in the recovery indicators, except for coronary diseases in females using fixed-effects and in males using pooled cross-sectional estimations (results shown in the supplementary online material). Results for the variable cardiovascular disease, representing all of the three related variables, are thus reported. Tables 3 and 4 show the results from the mediation analysis (for both genders), with the recovery indicator, *t2012*, reported as our main independent variable (full results from the base analysis are reported in the supplementary online material). Tables 5 and 6 for the pooled logit model in the sensitivity analysis are comparable to Tables 3 and 4 for the fixed-effects models. Figures 6 and 7 reveal the differences in the results for the recovery indicator, *t2012*, and indicator for short-term exposure, *t2009*, between the estimation models used. The figures show point estimates from regressions as well as 90% confidence intervals.

**Table 3:** Mediation analysis - males: Linear probability fixed-effects estimates

Dependent variable	Hypertension			Cardiovascular Disease		
	dy/dx	Robust SE		dy/dx	Robust SE	
Without mediators						
t2012	-0.0469	0.0284	*	-0.0092	0.0172	
BMI included						
t2012	-0.0468	0.0292		-0.0119	0.0177	
1 if underweight	-0.0112	0.0158		0.0096	0.0097	
1 if overweight	0.0044	0.0215		0.0083	0.0121	
1 if obese	0.0546	0.0426		0.0210	0.0182	
Alcohol included						
t2012	-0.0607	0.0292	**	-0.0093	0.0177	
1 if daily drinker	0.0537	0.1090		0.1070	0.0612	*
1 if frequent drinker	0.1300	0.0874		0.0928	0.0412	**
1 if seldom drinker	0.1100	0.0873		-0.0895	0.0412	**
1 if rare drinker	-0.0654	0.0808		0.0698	0.0373	*
Smoking included						
t2012	-0.0666	0.0275	**	-0.0137	0.0175	
1 if daily smoker	0.0556	0.0355		-0.0165	0.0166	
1 if weekly smoker	-0.0140	0.0240		-0.0476	0.0249	*
1 if seldom smoker	0.0134	0.0535		-0.0525	0.0250	**
Perceived status in society included						
t2012	-0.0605	0.0298	**	-0.0036	0.0178	
1 if much better	0.0260	0.0368		-0.0108	0.0208	
1 if considerably better	0.0143	0.0159		0.0181	0.0096	*
1 if somewhat better	-0.0011	0.0142		-0.0046	0.0080	
1 if somewhat worse	-0.0502	0.0244	**	0.0039	0.0072	
1 if considerably worse	-0.0769	0.0483		-0.0023	0.0190	
1 if much worse	-0.0695	0.0623		0.1330	0.0741	*
Stress included						
t2012	-0.0566	0.0300	*	-0.0022	0.0179	
pss	0.0063	0.0038	*	-0.0009	0.0018	
Unemployment included						
t2012	-0.0436	0.0296		-0.0073	0.0176	
1 if unemployed	0.0098	0.0451		-0.0097	0.0276	
Income included						
t2012	-0.0534	0.0285	**	-0.0148	0.0172	
real income	-0.0017	0.0038		0.0018	0.0015	
Working hours included						
t2012	-0.0726	0.0318	**	-0.0084	0.0193	
working hours per workday	-0.0016	0.0016		0.0005	0.0011	

Sample weights are applied. Covariates controlled for are number of children, marital status, residence, presciption mediation, and short-term crisis coefficient (t2009). *p < 0.1, **p < 0.05, ***p < 0.01

**Table 4:** Mediation analysis - females: Linear probability fixed-effects estimates

Dependent variable	Hypertension			Cardiovascular Disease		
	dy/dx	Robust SE		dy/dx	Robust SE	
Without mediators						
t2012	0.0739	0.0341	**	-0.0414	0.0145	***
BMI included						
t2012	0.0489	0.0326		-0.0391	0.0146	***
1 if underweight	0.0341	0.0352		-0.0068	0.0088	
1 if overweight	0.0222	0.0161		-0.0113	0.0094	
1 if obese	0.0283	0.0236		-0.0194	0.0117	*
Alcohol included						
t2012	0.0705	0.0347	**	-0.0434	0.0149	***
1 if daily drinker	-0.0239	0.0548		0.0152	0.0182	
1 if frequent drinker	-0.0610	0.0421		0.0106	0.0183	
1 if seldom drinker	-0.0461	0.0399		-0.0044	0.0178	
1 if rare drinker	-0.0408	0.0372		0.0032	0.0179	
Smoking included						
t2012	0.0730	0.0350	**	-0.0421	0.0149	***
1 if daily smoker	-0.0141	0.0349		0.0040	0.0069	
1 if weekly smoker	-0.0082	0.0452		0.0053	0.0062	
1 if seldom smoker	-0.0246	0.0348		0.0038	0.0036	
Perceived status in society included						
t2012	0.0632	0.0354	*	-0.0440	0.0149	***
1 if much better	-0.0034	0.0627		-0.0007	0.0050	
1 if considerably better	0.0184	0.0198		0.0025	0.0081	
1 if somewhat better	0.0095	0.0128		-0.0001	0.0047	
1 if somewhat worse	-0.0131	0.0145		-0.0050	0.0064	
1 if considerably worse	0.0335	0.0255		0.0084	0.0115	
1 if much worse	0.0537	0.0563		-0.0588	0.0548	
Stress included						
t2012	0.0871	0.0352	**	-0.0433	0.0151	***
pss	-0.0018	0.0034		0.0008	0.0017	
Unemployment included						
t2012	0.0661	0.0347	*	-0.0358	0.0123	***
1 if unemployed	0.0117	0.0247		0.0301	0.0166	*
Income included						
t2012	0.0808	0.0355	**	-0.0468	0.0161	***
real income	-0.0071	0.0038	*	0.0013	0.001	
Working hours included						
t2012	0.0874	0.0351	**	-0.0428	0.0154	***
working hours per workday	-0.0043	0.0016	***	-0.0006	0.0004	

Sample weights are applied. Covariates controlled for are number of children, marital status, residence, presciption medication, and short-term crisis coefficient (t2009). *p < 0.1, **p < 0.05, ***p < 0.01

**Table 5:** Pooled logit model estimations - Mediation analysis: males

Dependent variable	Hypertension			Cardiovascular Disease		
	dy/dx	Robust SE		dy/dx	Robust SE	
Without mediators						
t2012	0.0029	0.0119		0.0009	0.0023	
BMI included	^ε^	^ε^		^ε^	^ε^	
t2012	0.0049	0.0112		0.0012	0.0023	
1 if underweight						
1 if overweight	0.0497	0.0118	***	0.0005	0.0022	
1 if obese	0.2080	0.0315	***	0.0051	0.0046	
Alcohol included						
t2012	0.0021	0.0119		0.0009	0.0022	
1 if daily drinker	0.0555	0.0362		0.0225	0.0156	
1 if frequent drinker	0.0215	0.0165		0.0010	0.0030	
1 if seldom drinker	0.0403	0.0173	**	0.0003	0.0024	
1 if rare drinker	0.0348	0.0212		0.0057	0.0040	
Smoking included						
t2012	0.0024	0.0120		0.0012	0.0022	
1 if daily smoker	-0.0089	0.0147		0.0051	0.0036	
1 if weekly smoker	-0.0836	0.0148	***	-0.0068	0.0043	
1 if seldom smoker	0.0251	0.0440		-0.0058	0.0051	
Perceived status in society included						
t2012	0.0023	0.0120		0.0002	0.0022	
1 if much better	-0.0201	0.0322		-0.0072	0.0037	*
1 if considerably better	-0.0292	0.0137	**	0.0047	0.0035	
1 if somewhat better	-0.0033	0.0123		-0.0021	0.0024	
1 if somewhat worse	0.0124	0.0196		-0.0006	0.0029	
1 if considerably worse	0.0382	0.0327		0.0096	0.0062	
1 if much worse	-0.0058	0.0358		0.0315	0.0207	
Stress included						
t2012	-0.0009	0.0121		0.0004	0.0022	
PSS	0.0071	0.0029	**	0.0013	0.0006	**
Unemployment included						
t2012	0.0009	0.0121		0.0006	0.0023	
1 if unemployed	0.0738	0.0387	*	0.0037	0.0056	
Income included						
t2012	0.0021	0.0120		0.0001	0.0023	
real income	-0.0008	0.0022		-0.0010	0.0005	*
Working hours included						
t2012	0.0041	0.0119		0.0001	0.0021	
working hours per workday	-0.0014	0.0012		-0.0004	0.0003	*

Results are presented as marginal effects. Sample weights are applied. Covariates controlled for are age, age squared, number of children, marital status, residence, education, prescription medication, and short-term crisis coefficient (t2009). ^ε^Missing coefficient due to perfect predictability of underweight; hence optimal weight and underweight are combined in this estimation as a benchmark. *p < 0.1, **p < 0.05, ***p < 0.01

**Table 6:** Pooled logit model estimations - Mediation analysis: females

Dependent variable	Hypertension			Cardiovascular Disease		
	dy/dx	Robust SE		dy/dx	Robust SE	
Without mediators						
t2012	0.0258	0.0132	*	0.0007	0.0026	
BMI included						
t2012	0.0224	0.0127	*	0.0004	0.0020	
1 if underweight	-0.0235	0.0961		^ε^	^ε^	
1 if overweight	0.0606	0.0133	***	0.0002	0.0022	
1 if obese	0.1710	0.0196	***	0.0032	0.0026	
Alcohol included		0.0132				
t2012	0.0238	0.0224	*	0.0002	0.0021	
1 if daily drinker	-0.0830	0.0158	***	^σ^	^σ^	
1 if frequent drinker	-0.0365	0.0150	*	-0.0009 ^σ^	0.0027 ^σ^	
1 if seldom drinker	0.0068	0.0166		0.0010	0.0022	
1 if rare drinker	0.0185			0.0025	0.0025	
Smoking included						
t2012	0.0247	0.0133	*	0.0004	0.0020	
1 if daily smoker	-0.0358	0.0127	***	-0.0020	0.0022	
1 if weekly smoker	-0.0367	0.0440		-0.0053 ^δ^	0.0028 ^δ^	*
1 if seldom smoker	-0.0385	0.0351		^δ^	^δ^	
Perceived status in society included						
t2012	0.0260	0.0134	*	0.0005	0.0019	
1 if much better	-0.0354	0.0443		-0.0014	0.0054	
1 if considerably better	0.0013	0.0179		-0.0051	0.0023	**
1 if somewhat better	-0.0153	0.0131		-0.0025	0.0019	
1 if somewhat worse	-0.0112	0.0160		-0.0028	0.0020	
1 if considerably worse	-0.0123	0.0228		-0.0002	0.0031	
1 if much worse	-0.0048	0.0475		-0.0037	0.0025	
Stress included						
t2012	0.0258	0.0135	*	-0.0002	0.0020	
PSS	-0.0034	0.0029		0.0007	0.0004	
Unemployment included						
t2012	0.0256	0.0133	*	0.0004	0.0020	
1 if unemployed	0.0190	0.0284		0.0110	0.0081	
Income included						
t2012	0.0247	0.0134	*	0.0009	0.0020	
real income	-0.0014	0.0033		-0.0007	0.0006	
Working hours included						
t2012	0.0254	0.0135	*	0.0003	0.0021	
working hours per workday	-0.0033	0.0016	**	-0.0004	0.0003	

Results are presented as marginal effects. Sample weights are applied. Covariates controlled for are age, age squared, number of children, marital status, residence, education, prescription medication, and short-term crisis coefficient (t2009). ^ε^Missing coefficient due to perfect predictability of underweight; hence optimal weight and underweight are combined in this estimation as a benchmark. ^σ^Missing coefficient due to perfect predictability of daily drinker; hence daily drinker and frequent drinker are combined in this estimation. ^δ^Missing coefficient due to perfect predictability of seldom smoker; hence seldom smoker and weekly smoker are combined in this estimation. *p < 0.1, **p < 0.05, ***p < 0.01

### Fixed effects

A statistically significant (at the 10% level) negative relationship between the recovery indicator and hypertension in males was found, but a statistically -significant relationship was not found for cardiovascular diseases. For females, the recovery indicator is statistically significant for both hypertension and cardiovascular diseases (at the 5 and 1% level respectively), but the sign of the coefficient is not the same; a positive relationship is found between the recovery indicator and hypertension, but a negative relationship when cardiovascular diseases are explored. Point estimates for the recovery indicator reveal a decreased probability for females of having cardiovascular disease during the economic recovery by 4.14 percentage points compared to pre-crisis (Table 4). For hypertension, our estimates point to an increased probability of having hypertension during the economic recovery by 7.39 percentage points compared to pre-crisis for females (Table 4), but a decrease of 4.69 percentage points for males (Table 3).

In the mediation analysis for hypertension in males, the recovery indicator was consistently negative, and statistically significant with the addition of every mediator except BMI and unemployment. For females, the same consistency was found, with the recovery indicator remaining positive and statistically significant with the addition of all mediators except BMI. The addition of possible mediators resulted in both a reduction and an increase in the coefficient for the recovery indicator, *t2012*, leading to the conclusion that some of the possible mediators serve as mediators and some as suppressors, although generally not confirmed with mediation tests (*p* > 0.1). For convenience, we guide the reader through one mediator (unemployment) for hypertension in females and one suppressor (stress) (see Table 4). The coefficient for the recovery indicator in the base model is indicated at the top of the table (0.0739). When income is added to the model, the recovery indicator is reduced (0.0661) by 10.55%, which indicates that changes in unemployment explain 10.55% of the recovery effect on hypertension in females. Smoking, BMI, alcohol consumption, and a person’s perception of their financial status in society are also identified as mediators, albeit to a very limited extent and, except in the case of smoking, not confirmed with mediation tests (*p* > 0.1). The increased probability of females having hypertension between 2007 and 2012 seems suppressed by changes in stress and according to the mediation analysis hypertension would have increased by 17.86% more than current estimates suggest if no changes in stress would have occurred between waves. The other variables that were identified as suppressors for hypertension in females were working hours and income.

### Sensitivity analysis – pooled logit model estimations

No statistically significant recovery effect was linked to cardiovascular diseases for either gender. However, a statistically significant effect at the 10% level was found for hypertension in females. Point estimates for the recovery indicator reveal an increased probability of having hypertension during the economic recovery by 2.58 percentage points compared to pre-crisis. A statistically significant relationship was not found for males.

In the mediation analysis for hypertension, the recovery indicator was never statistically significant for males (Table 5), but for females (Table 6) it was significant for every addition of a mediator. For females the point estimates for the recovery indicator lowered with the addition of the variables representing health behaviors (BMI, alcohol consumption, and smoking) and the labor-market variables (unemployment, income, and working hours), with BMI mediating the largest effects out of the possible mediators studied (13.28%). However, stress and people’s perception of their relative financial status in society led to an increase in the recovery indicator, thus serving as suppressors in the relationship.

Results for the relationship between cardiovascular disease and the recovery indicator are reported in Tables 5 and 6 for males and females respectively. As expected, the precision of those measurements is low and the relationship is never found to be statistically significant, with or without mediators, for either gender.

When comparing results from our main analysis to the pooled logit models in the sensitivity analysis we find that the linear probability fixed-effects model shows a recovery effect that is larger in magnitude and higher in statistical significance for hypertension in both genders and for cardiovascular diseases in females (Figs. 6 and 7, and Tables 3, 4, 5 and 6).

### Sensitivity analysis – prescription medication

The results found when using prescription medication as the dependent variables paint a very similar picture as our main results, both when using fixed-effects models and pooled logit models in the estimations (see Tables 7 and 8). The sign of the coefficient for the recovery indicator is negative, and statistical significance is quite similar to our main results except in the case of hypertension in females where we do not find a statistically significant relationship when using prescription medication as a dependent variable (see Tables 3, 4, and 7). Predictably, a positive, highly statistical relationship is found between age and the use of prescription medication. Rather striking though, is the high statistical significance for the recovery indicator, especially when compared to our main results using diagnosis of the diseases as a dependent variable. The most obvious reason for this difference is that participants were asked about their usage of prescription drugs in the last 2 weeks, but when asked about a diagnosis of a disease no time perimeter was set in the questionnaires from 2007 to 2009 and it was 12 months in the questionnaire from 2012.

**Table 7:** Prescription medication usage in the last 2 weeks – fixed-effects model estimations

Dependent variable	Hypertension-medication			Cardiovascular & cholesterol-medication		
	dy/dx	Robust SE		dy/dx	Robust SE	
Males						
t2012	-0.0506	0.0226	**	-0.0303	0.0238	
t2009	-0.0157	0.0110		-0.0225	0.0100	**
Age squared	0.0003	0.0001	***	0.0002	0.0001	***
No. of children	-0.0030	0.0215		-0.0116	0.0200	
1 if rural	-0.0084	0.0189		-0.0378	0.0277	
1 if cohabiting	0.0061	0.0138		0.0054	0.0194	
1 if married	0.0217	0.0257		0.0312	0.0283	
1 if divorced	-0.0877	0.0733		0.0300	0.0387	
1 if widowed	-0.0743	0.0696		0.0224	0.0540	
n	4163			4260		
Females						
t2012	-0.0035	0.0227		-0.0564	0.0193	***
t2009	0.0076	0.0105		-0.0257	0.0088	***
Age squared	0.0001	0.0001	***	0.0002	0.0000	***
No. of children	-0.0239	0.0101	**	-0.0115	0.0049	**
1 if rural	0.0164	0.0195		0.0097	0.0140	
1 if cohabiting	-0.0004	0.0110		-0.0118	0.0113	
1 if married	0.0071	0.0182		-0.0075	0.0164	
1 if divorced	-0.0154	0.0311		0.0375	0.0264	
1 if widowed	0.0181	0.0431		0.0633	0.0566	
N	4790			4872		

Sample weights are applied. *p < 0.1, **p < 0.05, ***p < 0.01

**Table 8:** Prescription medication usage in the last 2 weeks – pooled logit model estimations

Dependent variable	Hypertension-medication			Cardiovascular & cholesterol-medication		
	dy/dx	Robust SE		dy/dx	Robust SE	
Males						
t2012	0.0265	0.0093	***	0.0143	0.0070	**
t2009	0.0153	0.0097		0.0025	0.0055	
Age	0.0238	0.0016	***	0.0130	0.0011	***
Age squared	-0.0002	0.0000	***	-0.0001	0.0000	***
No. of children	-0.0066	0.0028	**	-0.0037	0.0016	**
1 if rural	-0.0004	0.0070		-0.0021	0.0043	
1 if cohabiting	0.0245	0.0226		0.0206	0.0157	
1 if married	0.0017	0.0151		0.0002	0.0097	
1 if divorced	-0.0421	0.0127	***	-0.0111	0.0114	
1 if widowed	-0.0009	0.0205		0.0416	0.0259	
educ2	-0.0030	0.0087		-0.0092	0.0051	*
educ3	0.0291	0.0122	**	0.0028	0.0062	
educ4	0.0004	0.0124		-0.0142	0.0073	*
educ5	0.0038	0.0134		0.0090	0.0105	
N	4151			4250		
Pseudo R-squared	0.206			0.206		
Females						
t2012	0.0203	0.0105	*	0.0101	0.0034	***
t2009	0.0166	0.0107		0.0036	0.0027	
Age	0.0178	0.0021	***	0.0064	0.0007	***
Age squared	-0.0001	0.0000	***	0.0000	0.0000	***
No. of children	0.0025	0.0032		0.0002	0.0008	
1 if rural	0.0186	0.0091	**	-0.0007	0.0020	
1 if cohabiting	-0.0099	0.0209		-0.0007	0.0066	
1 if married	-0.0035	0.0169		0.0018	0.0045	
1 if divorced	-0.0098	0.0207		0.0094	0.0081	
1 if widowed	0.0092	0.0223		0.0050	0.0063	
educ2	-0.0214	0.0205		-0.0003	0.0054	
educ3	-0.0150	0.0099		-0.0026	0.0027	
educ4	-0.0327	0.0114	***	-0.0045	0.0034	
educ5	-0.0429	0.0142	***	-0.0074	0.0051	
n	4774			4856		
Pseudo R-squared	0.184			0.214		

Sample weights are applied. *p < 0.1, **p < 0.05, ***p < 0.01

In light of the attrition between the original sample and the final sample used in our analysis an attrition analysis was performed. This was also done in an attempt to understand better the reversal of the relationship between the shorter-term crisis and hypertension found by Asgeirsdottir et al. [7] using panel estimations and the differing results from the methods used here. By comparing means between groups we found that there are more non-attritors who report having hypertension in general than attritors, but fewer who report having developed hypertension during the economic collapse, between 2007 and 2009. Our main internal validity concern is that participants who reported having developed hypertension or cardiovascular diseases in the years between 2007 and 2009 had attrited and were thus not a part of the sample in 2012. This was indeed found to be the case for cardiovascular diseases, but this hypothesis was however rejected in our attrition analysis in the case of hypertension (result available in the supplementary online material). However, the sign of the coefficient is comparable in the attrition analysis between genders, thus not explaining the differing results found in our analyses.

## Discussion

A priori the recovery effects under examination here are not known, where one could well imagine that health effects influenced by changes in the economy could diminish or even disappear with the stabilization of economic conditions. Conversely, some diseases take time to emerge, e.g. because of persistent exposure to stressful circumstances caused by ambient economic conditions. Cardiovascular diseases have both elements of cumulative build up, as well as sensitivity to immediate circumstances.

The results found using fixed-effects models and pooled logit models were consistent across some dimensions while conflicting across others. When effects were found for females, they consistently showed hypertension to be greater during the recovery period than the boom. However, while fixed-effects estimations revealed statistically significant results for both genders (in opposite directions), statistically significant results were found only for females using pooled logit models. Furthermore, pooled cross-sectional estimations reported in the supplementary online material showed statistically significant results for men only. Our sensitivity analysis using prescription medication as a dependent variable supported our main results to a large extent, with the exception of hypertension in females where the recovery indicator was not statistically significant. A priori we did not expect such similarities to emerge since the definition of the outcome variables are quite different, as is the time frame for each one (participants were asked about their use of prescription medication in the last two weeks, but when asked about the diagnosis of a medical condition the time frame indicated in the questionnaires from 2007 to 2009 was simply “in the past” and in the 2012 questionnaire it was changed to “the last 12 months”). As people’s memory can become less reliable as time goes by, the accuracy in answers is arguably better with a shorter time-frame for participants to consider, but on the other hand not all who are diagnosed with a disease decide to use medication to combat the disease. Therefore it is not obvious which variable better captures what we want to measure – the long-term exposure of the economic collapse on health. Thus it’s important to view the results from the primary estimations and sensitivity analysis together as a whole.

Previously published results by Asgeirsdottir et al. [7] showed statistically significant hypertension effects of the crisis in the short term in males only. Our results using pooled data in a similar fashion as they did showed a different timing of responses across genders; with males showing a more immediate response and dwindling with time (not statistically significant), and females showing a delayed response during the economic recovery as opposed to the height of the crisis in 2009.

Although our results using fixed-effects models point to a negative relationship between long-term exposure to the crisis (recovery indicator) and cardiovascular diseases (statistically significant for females), those results were found to be affected by attrition. Perhaps not surprisingly, those results were not found to be stable across estimation strategies, with no statistically significant relationship found using pooled logit models. Those results are though reported for completeness as was done in the study on short-term effects of the crisis [7].

Not all our results are robust to changes in the estimation model used. However, the coefficient that remained stable and statistically significant was the recovery indicator when estimating hypertension in females. Furthermore, bias due to attrition was not found to be present in those estimations. Our main conclusion is thus that during the economic recovery in 2012, when the dust was settling after the economic collapse, Icelandic women had an increased probability of having hypertension compared to the boom year 2007.

In light of the commonalities of our research and that of Asgeirsdottir et al. [7], the causes of the differences in results are worth further attention and even further exploration in future research. As mentioned earlier, the nature of diseases can vary. This could explain why the elevated hypertension during the recovery period in men is no longer found when a balanced panel is used, but has instead appeared in women. However, this would not explain why the gender effects are reversed when new individuals in both 2007 and 2012 are studied, as found in the cross-sectional estimations reported in the supplementary online material. If death of males who previously reported having hypertension is the main cause of the altered results between years, or the different results found between estimation methods, that in itself would be noteworthy, but results for the attrition analysis did not confirm a systematic attrition of males in particular. Information on the fate of individual participants is however not accessible at this time, barring us from that line of research.

This later-time appearance of a female response in the panel data is also interesting as the male-only effect in the previous study had been somewhat puzzling, especially in light of research showing a stronger short-term stress response to the crisis in females than in men using the same data as we examine (waves from 2007 to 2009), where the male stress response was largely measured without statistical significance [5]. Similarly a female-only result was reported for the change in attendance at cardiac emergency departments in Reykjavík, Iceland immediately following the economic collapse in October 2008, which was not observed at other emergency departments [8]. Even further, misuse of alcohol had been reported to go down to a greater extent for males than for females [2]. Those are all results that would suggest a greater effect on female hypertension and CVD than on males, which made the previous results puzzling. The current findings may indicate a lingering female response that may have taken longer to come through. That would be in line with some previous findings, although it has to be kept in mind that the found effects could also be the immediate result of a growing economy in 2012, rather than a delayed effect of the crisis.

This study has both strengths and limitations. The main strengths lie in the comparability to the study by Asgeirsdottir et al. [7], providing additional information on the fates of the same individuals under study using both a pooled logit model as they did as well as using a balanced panel to examine individual fixed effects. Additionally, in the supplementary online material we report results for the pooled cross-sectional estimations, and thus results for always-in-participants and new participants in 2012 can be compared and the possibility of a selection bias in the always-in sample is dealt with. Furthermore, in the supplementary online material we include a mediation analysis using fixed effects which allows for the possibility of moderated mediation. In such a model one could hypothesize various interactions and pathways; marriage may provide some risk sharing, the presence of children in the household could give people less flexibility in adjusting to the crisis, for example by relocating for a different job. Although the current approach is kept in line with the previously published literature, we have included an example of one such pathway, where we add an interaction term of the recovery indicator and the possible mediating factor. Further exploration of this type is a possible avenue for further research.

Moreover, a notable strength is the health outcomes chosen to explore, cardiovascular morbidity, that are available at an individual basis, but most previous studies have used aggregate mortality data, both disease-specific mortality [44, 83] and overall mortality [38, 42, 44, 84]. Death is the severest outcome, and only focusing on that can mask some real health effects that do nevertheless affect people’s lives. The study adds to the literature, although results cannot be directly compared with those studies utilizing mortality outcomes.

The analysis by Gudlaugsson et al. [66] of the latest data in *Health and well-being of Icelanders* shows that the health of young Icelandic women, as reported by themselves, has deteriorated across the spectrum in the period between 2007 and 2012. This applies to both mental and physical conditions. The opposite was found for young males, who generally reported better health in 2012 than 2007. These results complement those found in our panel estimations, but also raise questions on why changes in health are materializing differently for males and females. The analysis by Gudlaugsson et al. [66] uses the full dataset available, i.e. all 2012 participants regardless of whether they are new to the sample or not (as well as those who did not fulfil our specifications) and is not in accordance with our results using new entrants in 2012 (results in the supplementary online material), where the economic-recovery indicator shows a stronger association to hypertension in males than females. Although we find a consistently positive link between long-term exposure to the crisis into the recovery period and hypertension in females, both methods used here reveal an unexplained difference in the size of the recovery coefficient between genders. Although fluctuations in the prevalence of hypertension of Icelanders (see Fig. 3) could theoretically be an explanation for our findings, our limited analysis of patterns in related data on hypertension suggests otherwise. First and foremost, a steady decline in the prevalence of hypertension among cardiovascular patients after 2008 suggests that the economic collapse had a beneficial impact on that specific patient group; Icelanders suffering from hypertension were not admitted to the hospital because of cardiovascular diseases to the same extent as before. Given the lack of available data on overall prevalence of hypertension in Icelanders, the aggregate data on cardiovascular patients at Landspitali University Hospital probably gives the strongest clue on the true incidence of hypertension in Iceland. Furthermore, predicted hypertension prevalence, drug use, and circulatory-disease mortality suggest that our findings for females represent a deviation from pre-crisis trends and thus signify a true longer-term crisis impact. Further research is well warranted on that issue.

## Conclusions

We find that during the economic recovery in 2012, Icelandic women had an increased probability of having hypertension compared to the boom year 2007. For males, the results were more ambiguous. This study adds to the strand of literature concerning the relationship between economic cycles and health. Results from other studies regarding this relationship are mixed between settings, and thus our results conform to some while being conflicting to others. We provide results based on individual-level morbidity data, whereas the literature mostly contains studies using mortality data due to data restrictions. The small size of the Icelandic economy might diminish the generalizability of our results, but having said that, the country is a western country, in which the health-care system and health status rival most western societies and standards of living are also comparable. This leads us to conclude that the generalizability and comparability of our results are fairly strong.

## Abbreviations

BMI: Body mass index
CVD: Cardiovascular disease
ISK: Icelandic kronas
PSS: Perceived stress scale
